# Deciphering salt tolerance mechanisms in synthetic hexaploid and bread wheat under humic acid application: physiological and genetic perspectives

**DOI:** 10.3389/fpls.2025.1545835

**Published:** 2025-03-06

**Authors:** Fahad Alghabari, Zahid Hussain Shah

**Affiliations:** ^1^ Department of Arid Land Agriculture, King Abdulaziz University, Jeddah, Saudi Arabia; ^2^ Department of Plant Breeding and Genetics, PMAS-Arid Agriculture University, Rawalpindi, Pakistan

**Keywords:** synthetic hexaploid, gene regulation, antioxidant, correlation, heatmap

## Abstract

Salt stress is a potential constraint that perturbs plant physiological and osmolytic processes, and induces oxidative stress. The plant biostimulant, such as humic acid (HA) is capable to improve the wheat-tolerance to salt stress through triggering the plant defense mechanisms and regulating the genetic determinants. In this context the present study has comparatively evaluated the effect of HA on salt tolerant synthetic hexaploid (SH) and salt susceptible bread wheat (BW) genotypes. The experiment was performed in three replicates using randomized complete block design (RCBD) having two factorial arrangements, with HA treatment as one, while genotype as second factor. HA treatment significantly enhanced chlorophyll (33.33%–100%) and photosynthesis (31.25%–50%), and significantly reduced the glycine betaine (GB) (42.85%–77.77%), proline (20%–28.57%) and Na^+^/K^+^ ratio (33.33%–50%) in salt stressed SH and BW genotypes. Additionally, HA significantly increase the activities superoxide dismutase (SOD), peroxidase (POD), and catalase (CAT) by 57.14%–66.67%, 54.54%–83.33%, and 55.55%–80%, respectively in all salt stressed genotypes. The salinity associated genes *TaNHX1, TaHKT1,4, TaAKT1, TaPRX2A TaSOD* and *TaCAT1* were upregulated, while *TaP5CS* was downregulated in SH and BW genotypes corresponding to their regulatory traits. Furthermore, the multivariate analysis including correlation, principal component analysis (PCA) and heatmap dendrogram further rectified the strong impact of HA on the strength of association and expression of stress marker traits. Overall, the SH genotypes showed more strong response to the HA and illustrated significant tolerance to salt stress based upon physiological, biochemical and genetic indicators. Conclusively, the SH can serve as a bridge to transfer alien genes associated with salt tolerance into elite bread wheat germplasm.

## 1 Introduction

Wheat is an important cereal crop dominating the most of the arable land worldwide. The stressful environment can decrease wheat production by 6% as predicted by different climatic models (Teleubay et al., 2024). Salt stress imposes negative impacts on 20% of world arable land owe to anthropogenic activities and the change in climate (El Sabagh et al., 2021). Approximately 1.4 billion hectare area of the world is effected by salinity (FAO, 2024). The saline environment imposes osmotic stress, ionic imbalance and toxicity, through high Na^+^ influx that increases Na^+^/K^+^ ratio and retards the plant vital processes and crucial functions (Shah et al., 2017). Besides, salt stress brings variation in ultrastructural components of cells, damages the membrane, perturbs the photosynthesis machinery, enhance the oxidative stress through reactive oxygen species (ROS) which limits the plant ultimate productivity (Sachdev et al., 2021). In depth understanding of stress tolerance in plants with respect to physiological mechanisms is an important breeding goal (Chaudhary et al., 2024). Therefore, the concept of physiological, biochemical and genetic mechanisms of wheat responses under salt stress is inevitable. Among physiological features, the loss of photosynthetic pigments, while among biochemical features the accumulation of osmolytes is the focal point of plant research particularly under salt stress (Gupta and Huang, 2014). Moreover, plants exposed to salt stress undergo various oxidative changes like production of reactive oxygen species (ROS), lipid-peroxidation and plasma membrane deterioration (Hasanuzzaman et al., 2021). Being living body plant try to retain its homeostasis through activating the antioxidant system having enzymes such as peroxidases (PODs), superoxide dismutases (SODs) and catalases (CATs) (Mehla et al., 2017). Therefore, an understanding of physiological and biochemical patterns of salinity tolerance can be used as a selection parameter for improving the wheat adaptability in saline environment (Irshad et al., 2022). In past many studies have proved strong link between physio-chemical and genetic responses of wheat under different kinds of abiotic stresses (Yang et al., 2014; El Sabagh et al., 2021; Alsamadany et al., 2023; Alghabari and Shah, 2024). Briefly, plant behave like a composite system to sustain their physio-chemical equilibrium through modulating their osmotic and genetic responses when exposed to salt stress (Shah et al., 2017). Besides, for comprehensive elucidation of salt tolerance mechanisms in wheat, it is important to explore the physio-chemical responses of plant in association with their genetic determinants (Gupta and Huang, 2014). For instance, the upregulation of genes *TaCAT1, TaSOD* and *TaPRX2A* in salt stressed wheat plants triggers the antioxidant activities and increases the ROS scavenging via activating the antioxidant defense system comprising the enzymes CAT, SOD and POD (Alghabari and Shah, 2024). Similarly, *TaP5CS* gene increases the proline accumulation in wheat during salt, drought and heat stresses (Aycan et al., 2022). High affinity K^+^-transporters (*HKTs*), and Na^+^ proton-exchangers (*NHXs*) are membrane transporters that respectively regulate K^+^-influx and Na^+^-efflux, therefore reducing the Na^+^ toxicity through decreasing Na^+^/K^+^ ratio (Hussain et al., 2021). Likewise, an inward-rectifying K^+^-channel, *AKT1* increases plant tolerance against osmotic-stress by increasing the concentration of K^+^ in cell as reported by Ahmad et al. (2016). Salinity stress impairs wheat growth and yield by causing osmotic stress, ionic imbalance, and osmotic damage (Ali et al., 2022). HA overcomes these effects by increasing chlorophyl, photosynthesis and antioxidant enzyme activities as reported by Tahir et al. (2022) and Meng et al. (2023). Furthermore, it also regulates the osmolytic level, improves the osmotic adjustments and salt tolerance (Zhang et al., 2024). Besides, HA improves the Na^+^/K^+^ ratio in salt stressed plants. Meng et al. (2023) reported that HA reduces the proline accumulation in salt stressed plants, indicating that HA alleviates the osmotic stress, hence decreasing the need of proline as an osmoprotectant. Likewise, HA application is associated with decrease in GB content salt stressed plants, suggesting improvement in water status and homeostasis, thereby minimizing the necessity of GB accumulation (Carillo et al., 2008). Various studies in past have been conducted to elucidate the potential role of HA in enhancing the salt tolerance of wheat (Carillo et al., 2008; Ali et al., 2022; Tahir et al., 2022; Meng et al., 2023). The plant biostimulants such as humic acid (HA) plays executive role in maintaining plant physiological activities during salt stress through increasing the production of osmoprotectants, and through regulating the expression of genes imparting stress tolerance (Ahmad et al., 2022; Meng et al., 2023). Reduced sodium toxicity, osmoprotectant regulation, a decrease in the Na+/K+ ratio, and an increase in antioxidant enzyme activity are all linked to HA-induced salt tolerance, which in turn lowers oxidative stress levels (Shukry et al., 2023). Although various studies have explored the aspects of salinity tolerance by integrating the physiological, biochemical, and genetic mechanisms, the present study aimed to elucidate the impact of HA on synthetic hexaploid (SH) wheat as compared to bread wheat (BW). Furthermore, this study supersedes the past researches by not only exploring the physiological, biochemical and genetic indices of salt tolerance but also providing a comparative analysis between wheat types, thereby providing new perceptions into their varying responses to HA under salt stress.

## 2 Materials and methods

The present experiment was performed within the glass house situated at the research site of the King Abdulaziz University (KAU), Jeddah, Saudi Arabia. The wheat genotypes shown in **Table 1**, including salt susceptible bread wheat cultivars, Kohistan-97, Fareed-06, A.Sattar (Irshad et al., 2022) and salt tolerant synthetic hexaploid (Li et al., 2018) were collected from Pakistan and evaluated for physiological, biochemical and genetic performance under saline condition in the presence and absence of HA. The experiment was executed in two factorial tri-replicated RCBD with HA and control treatments as one, and genotypes as another factor.

**Table 1:** Wheat germplasm containing synthetic hexaploid (SH) and salt susceptible bread wheat (BW) genotypes.

Genotype	Pedigree
Synthetic Hexaploids (Salt tolerant)	
SH1	TC870344/GU1//TEMPORALERA M 87/AGR/3/WBLL1
SH2	GPO8 KAZAKSTAN 6 WM98-99/4/KAUZ//ALTAR 84/AOS/3/KAUZ/5//KAUZ//ALTAR 84/AOS/3/KAUZ
SH3	CROC_1/AE.SQUAROSSA (205)//BORL95/3/KENNEDY
SH4	PAM94/3/ALTAR84/AEGILOPS SQUAROSA(TAUS)//OPATA/PASTOR
Bread Wheat (Salt suceptible)	
Kohistan-97	V-1562//CHIROCA(SIB)/(SIB)HORK/3/KUFRA-I/4/CARPINTERO(SIB)/(SIB)BLUEJAY[1964][2857]
Fareed-06	PTS/3/TOB/LFN//BB/4/BB/HD-832-5//ON
A. Sattar	PRL/PASTOR//2236(V.6550/Sutlej-86

### 2.1 Plant growth and treatment application

The tri-replicated experiment was optimized in glass house (25/15 ± 2°C day/night temperature, 10 hour light-period, 60% humidity) using 2.5liter pots, having 30cm depth and 25 cm diameter. The pots were filled with natural soil (loamy soil with pH_6.5, EC 1.5 dS/m) and sown with six seeds, while each pot was left with four plants at seedling stage. The experimental set of pots was provided with HA (Dalian Vic Co., Ltd., Dalian, China) having specified composition (C=55%, P=0.01%, N=4.87%, K=11.2%, Ca=0.50%, Mg=0.22%). The HA was applied at 2 g kg^−1^ before sowing of seeds (Khaled and Fawy, 2011; Canellas et al., 2015). The pots were watered twice a week by 500 ml of 25% Hoagland nutrient solution (Sigma-Aldrich, USA). The pot was watered with150 mM of NaCl solution that set the EC value at 8.5 dS/m. The EC values were monitored on daily bases using the conductivity meter.Both control and experimental set of plants were subjected to salt stress at tillering stage 5 (Feekes growth scale) until the symptoms of saline stress indicators for instance leaf wilting, rolling and chlorosis. Besides, each treatment comprised of five pots, while for data analysis five plants were selected on random basis for each treatment of every replicate.

### 2.2 Quantification of physiological and osmolytic traits

The SPAD-502plus apparatus was used to quantify the chlorophyll content, while the photosynthesis rate was recorded using infra-red gas analyzer (IRGA) apparatus (ADC Bioscientific, UK) from attached flag leaf between 9am to 10am. The Na^+^ and K^+^ content were estimated according to the procedure described by Havre (1961). In short, the leaf samples were oven dried and converted to fine powder. Afterward, 0.5g of each sample was homogenized in 3ml HClO_4_ and 8ml Conc HNO_3_ and retained for 12h. The mixture was burnt at 300°C for 3h and double distilled water was supplemented till 50ml volume. The flame photometer was used to record the concentrations of Na^+^ and K^+^. Before taking measurements, the flame-photometer was standardized and properly calculated by using known K^+^ and Na^+^ standard solutions prepared from high purity KCl and NaCl. With a blank solution the instrument was set at zero, and a calibration curve was structured by recording the emission intensities of standard solutions. After calibration the flame photometer was used to measure the Na^+^ and K^+^ concentrations from prepared samples, and Na^+^/K^+^ ratio was estimated accordingly. Furthermore, the UV-Vis spectrophotometer (N5000, Shanghai, China) was used to record the proline concentration based on its reactivity with ninhydrin as explained by Carillo and Gibon (2011). The glycine betaine (GB) content was determined using the HPLC apparatus (Shimadzu Corp, Japan) following the protocol explained by Grieve and Grattan (1983).

### 2.3 Quantification of antioxidant activity

For the quantification of antioxidant enzyme activity, 2g of frozen leaf sample was homogenized in 2mL of 0.1M Tris-HCl (pH=7.4) at ice cold stage. The mixture was optimized at 4°C and subjected to centrifugation for 20 minutes at 20000 g. The supernatant attained was used to record the enzymatic activity based on the standard protocol explained by Alici and Arabaci (2016). The activity of superoxide dismutases (SODs) was assessed against the standard absorption curve of 560nm based upon its tendency to inhibit the photo-reduction of nitroblue-tetrazolium. Moreover, the activity of peroxidases (PODs)was recorded by spectrophotometer against the standard absorption curve of 420 nm using 4-methylcatechol as substrate. The catalases (CATs) activity was measured using spectrophotometer at 25°C against standard absorption curve of 240nm, using H_2_O_2_ as substrate. Moreover, the catalytic activities of all antioxidant enzymes were measured in enzyme units (*U mg^-1^of protein).*

### 2.4 Gene expression analysis

The gene expression analysis was performed when plants showed the salt stress symptoms. The leaf samples from selected plants were instantaneously froze in a container having liquid nitrogen and stored at -80°C until the start of extraction process. The RNeasy kit (Qiagen, Germany) was used for RNA extraction according to the instructions of manufacturer. Besides, cDNA library was constructed from 2 µg RNA using QuantiTect reverse transcription kit (Qiagen, Germany). Furthermore, the transcript levels of salinity related genesin leaf tissue were quantified in qRT-PCR using SYBR Green Kit (BioFACT, Korea), while *TaActin1-*expressing gene was used for the expression normalization. Each expression profile from qRT-PCR analysis was analysed and confirmed following the procedure opted by Ahmed et al. (2022) using each of three biological and technical replicates. Furthermore, double-delta Ct values were used for the calculation of relative gene expression from each sample. The list of genes associated with salinity tolerance in wheat along with their forward and reverse primers is indicated in **Table 2**.

**Table 2:** The list of primers used in relative expression analysis of salt stress related genes in synthetic hexaploid (SH) and bread wheat (BW) genotypes.

Gene	Primer sequence	Reference
*TaAKT1*	CGGATAATGCCGTGAATG (F) TTATACTATCCTCCATGCCT (R)	Zeeshan et al., 2020
*TaCAT1*	TCTCTCGGCCAGAAGCTCG (F) AGGGAAGAACTTGGACGGC (R)	Al-Ashkar et al., 2019
*TaPRX2A*	CGTGTGTGTGATCATCAGTAAC (F) AGGCGGAGTGTAAATTACAAGA (R)	Su et al., 2020
*TaHKT1,4*	AGCAAGCTGAAGTTGAGGGG (F) AGAGTTGTGACAGAGCCGTG (R)	Dave et al., 2022
*TaP5CS*	GAAGGCTCTTATGGGTGTACTCAA (F) TAAAAGACCTTCAACACCCACAGG (R)	Aycan et al., 2022
*TaSOD*	TCCTTTGACTGGCCCTAATG (F) CTTCCACCAGCATTTCCAGT (R)	Jiang et al., 2019
*TaNHX1*	TGACGGAGGCAGAAGACCG (F) CCCAAAACTCTACACAGCGT (R)	Al-Ashkar et al., 2019

### 2.5 Data analysis

The Analysis of variance (ANOVA) was carried out using a computer-based statistics tool, Statistix ver. 8.1 (Mcgraw-Hill, 2008), at the probability level of 5% and LSD was computed, while the RStudio version 1.1.456 (RStudio Team, 2020) was applied to compute correlation, to perform principal component analysis (PCA), and to construct heatmap clusters based upon similarity matrix. The PCA biplots were constructed using “factoextra” and “FactoMineR”, while the “GGally” and “ggplot2” were used to form Pearson’s correlation chart. The packages “pheatmap” and “complex Heatmap” were used to form dendrogram.

## 3 Results

### 3.1 Chlorophyll, photosynthesis and osmolytes

**Figure 1:**
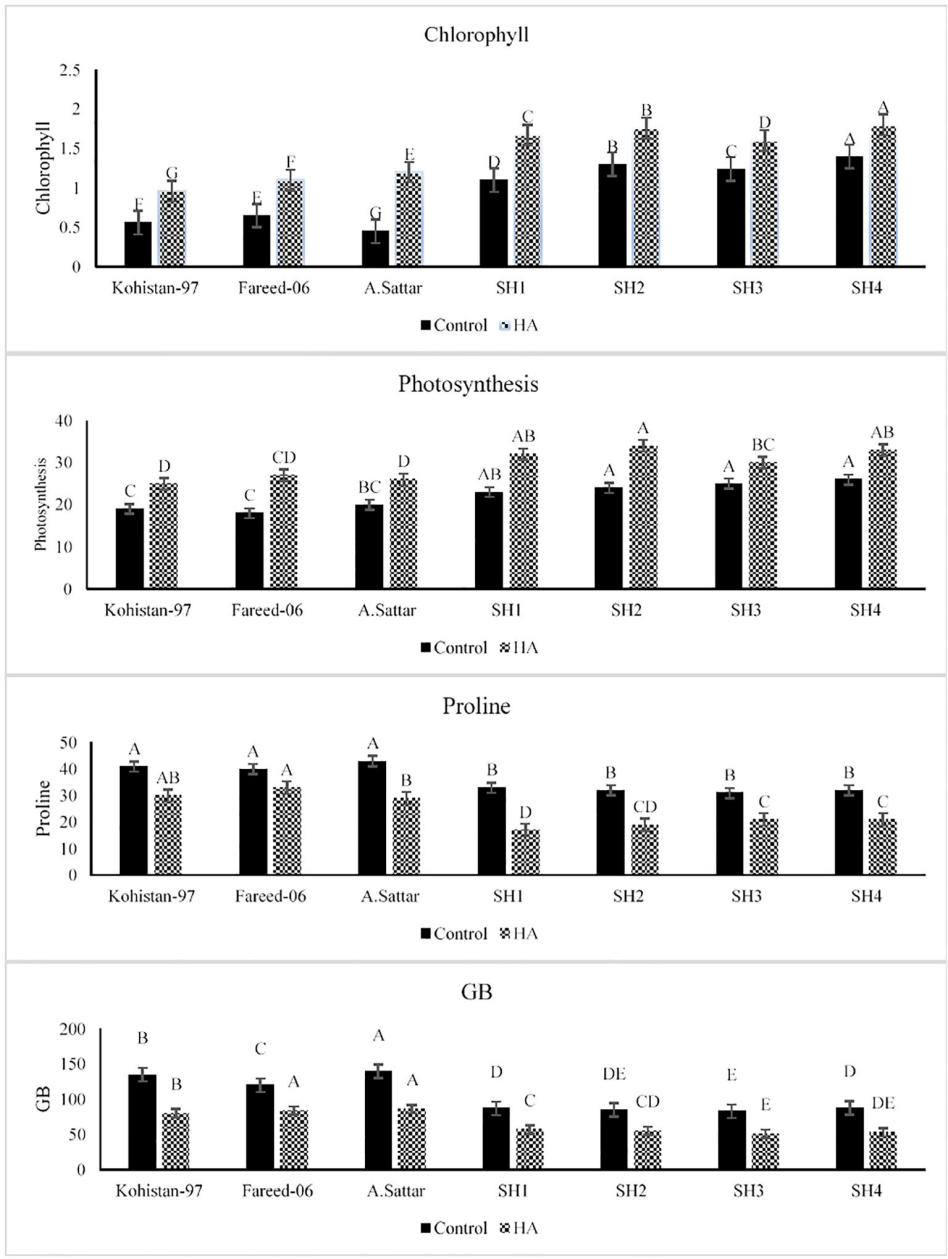
Effect of HA and control treatment on chlorophyll, photosynthesis, proline and glycine betaine content of different wheat genotypes grown under salt stressed environment. Chl, chlorophyll; Pn, photosynthesis; GB, glycine betaine. Graph bars represent the mean values of traits, analysed during tri-replicated two factorial experiment at p ≤ 0.05. The bar values following the different letters are significantly different at p ≤ 0.05.

The application of HA has significantly (p ≤ 0.05) improved the chlorophyl and photosynthesis rate (Pn) in all salt stressed wheat genotypes as compared to no application of HA (**Figure 1**). The synthetic hexaploid (SH1 to SH4) depicted superior physiological performance under HA application compared to other bread wheat (BW) cultivars (Kohistan-97, Fareed-06, A. Sattar). The chlorophyll content illustrated significantly higher baseline in SH1 to SH4 and increased 21.4%-50%, whereas Kohistan-97 showed a highly variable response with an 80% rise in chl content. Besides, photosynthesis rate (Pn) increased by 28%-36% in SHs (SH1 to SH4) that revealed more stable and higher assimilation of CO_2_ compared to sharper and inconsistent increase (42.8%-50%) in BW cultivars. Besides, under HA application, all salt stressed wheat genotypes showed significant (p ≤ 0.05) decline in their proline and glycine betaine (GB) contents as compared to set of wheat genotypes grown under control condition (**Figure 1**). Conversely the proline accumulation declined from 28.5%-50%, while the GB content declined from 50%-72%, in salt stressed SHs (SH1 to SH4) due to HA as compared to control. Moreover, unlike Kohistan-97, Fareed-06, and A. Sattar, which showed dramatic fluctuations, the SH1 to SH4 kept higher and stable physiological responses and less variation in osmoprotectants (proline and GB) under salt stress due to application of HA.

### 3.2 Antioxidant enzymes and Na^+^/K^+^ ratio

**Figure 2:**
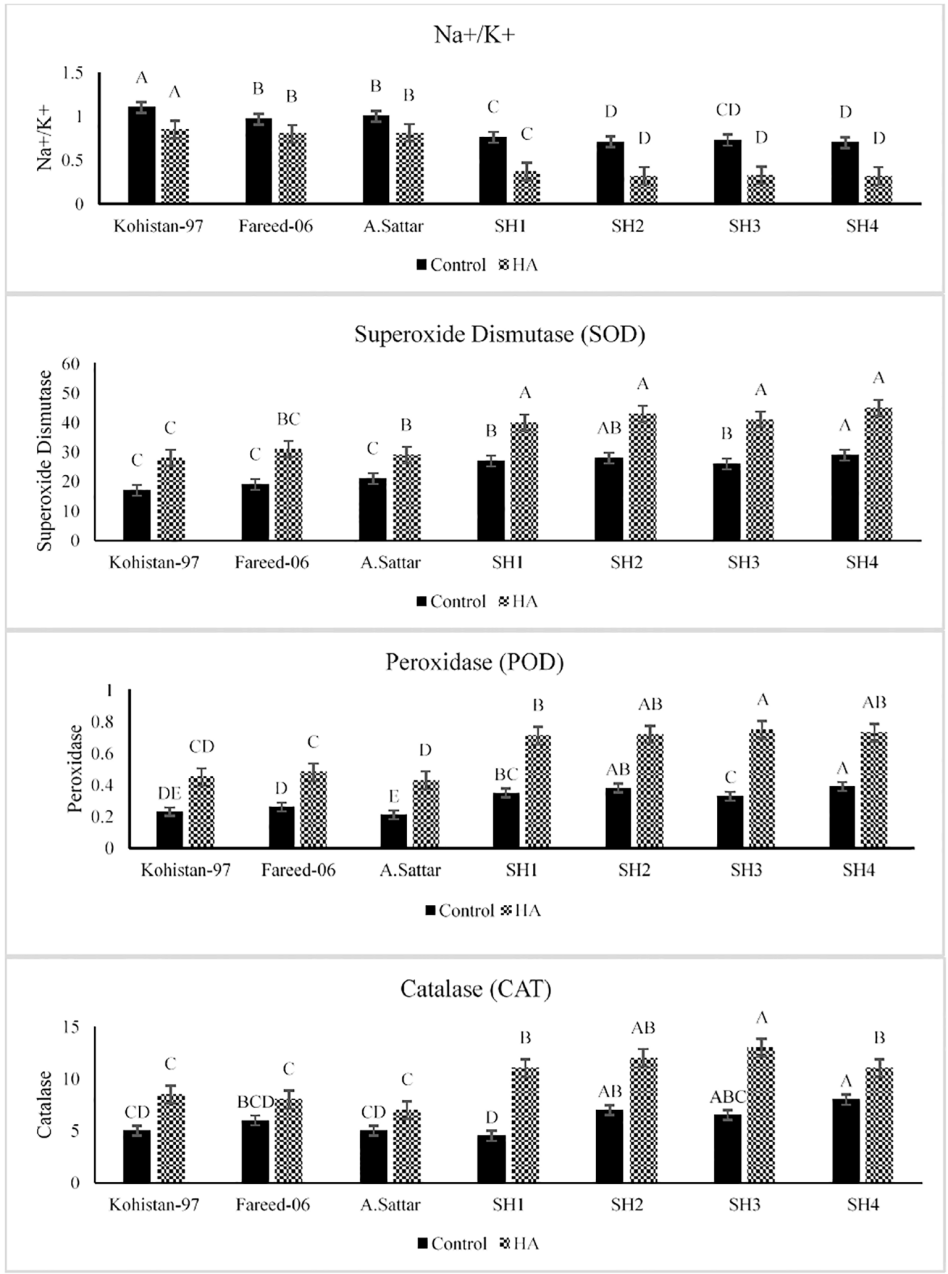
Effect of HA and control treatment on the activities of antioxidant enzymes (SOD, POD, and CAT) and Na^+^/K^+^ ratio of different wheat genotypes grown under salt stressed environment. SOD, superoxide dismutase; POD, peroxidase; CAT, catalase. Graph bars represent the mean values of traits, analysed during tri-replicated two factorial experiment at p ≤ 0.05. The bar values following the different letters are significantly different at p ≤ 0.05.

The HA caused significant (p ≤ 0.05) reduction in Na^+^/K^+^ ratio in all salt exposed set of plants as compared to the control set of plants (**Figure 2**). The SHs (SH1 to SH4) depicted significant reduction in Na^+^/K^+^ ratio 30% to 33.3% than BW cultivars Kohistan-97, Fareed-06 and A.Sattar 15% to 18.1% under saline conditions due to the application of HA, suggesting better ionic regulation and salt stress mitigation. Additionally, HA stimulated the activity of antioxidant enzymes catalase (CAT), superoxide dismutase (SOD), and peroxidase (POD) in all salt stressed wheat genotypes as compared to zero treatment of HA (**Figure 2**). The antioxidant enzymes superoxide dismutase (SOD) demonstrated increased catalytic activity by 20.8%-25%, in SH1 to SH4 as compared to 20% 26.6% in BW cultivars, suggesting their stable antioxidant defense system. Besides, the peroxidase (POD) activity increased by 27.2%-50% in SHs (SH1 to SH4) indicating better ROS scavenging compared to 42.8%-60% in BW genotypes. Furthermore, the catalase activity increased by 25%-44.4% in SH1 to SH4 explicating the better oxidative control under salt stress due to HA. Compared to Kohistan-97, Fareed-06, and A. Sattar, which manifested high variation in enzymatic activities, the SHs (SH1 to SH4) kept better Na^+^/K^+^ balance, consistent antioxidant response, and superior adaptability against salt stress under HA application.

### 3.3 Multivariate analysis

**Figure 3:**
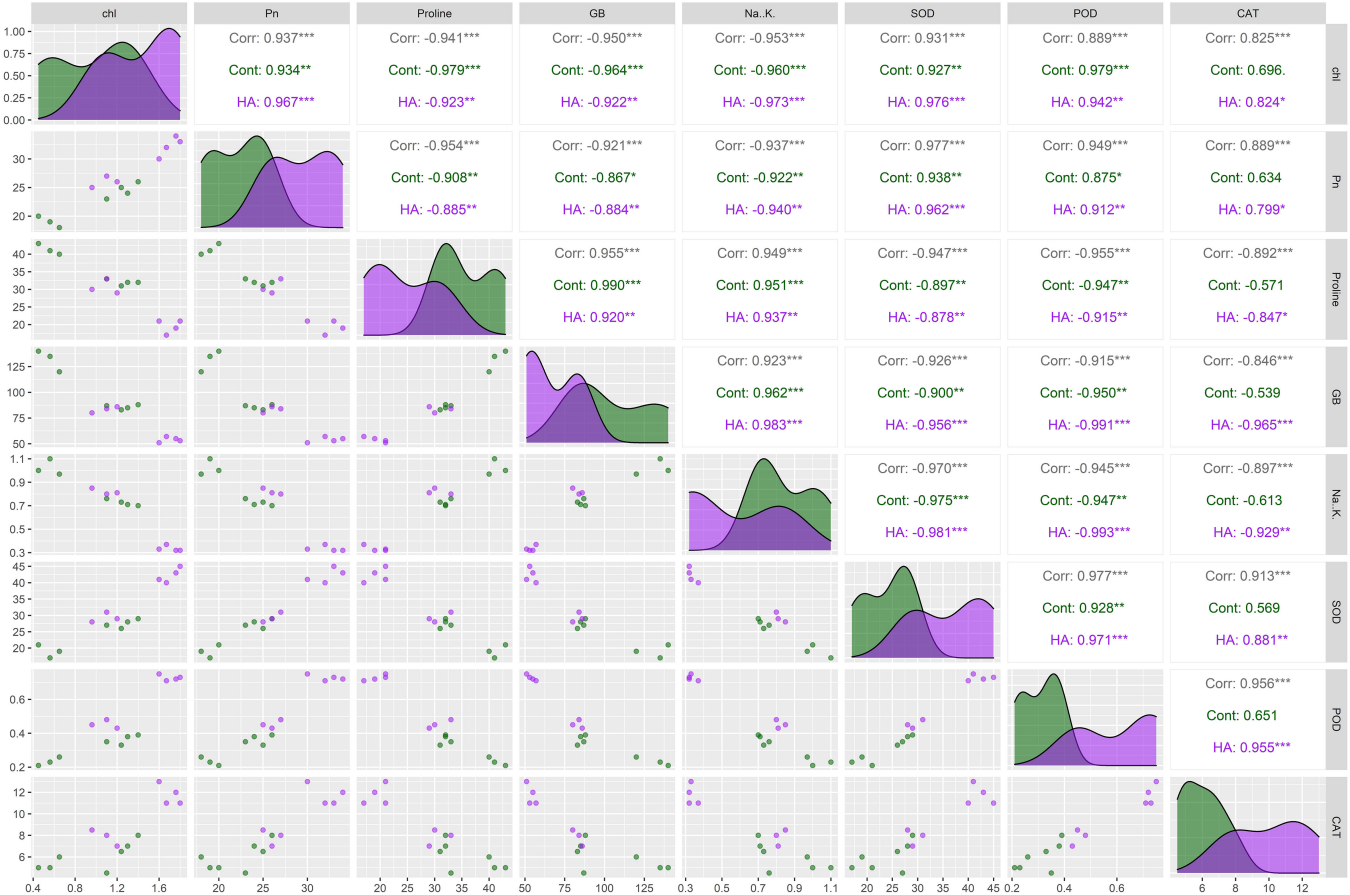
Correlogram showing the effect of control and HA treatment on the paired association of chlorophyll, photosynthesis, osmolytes (Proline and GB), Na^+^/K^+^ and antioxidant enzymes (SOD, POD and CAT) in salt stressed wheat genotypes. Chl, chlorophyll; Pn, photosynthesis; GB, glycine betaine; SOD, superoxide dismutase; POD, peroxidase; CAT, catalase. ***Significant at p ≤ 0.001, **Significant at p ≤ 0.01.

The correlogram revealed significant correlation among chlorophyll, Pn, proline, Na^+^/K^+^, GB, CAT, POD and SOD in all salt stressed wheat genotypes (**Figure 3**). The proline, GB and Na^+^/K^+^ illustrated showed significant positive paired association with one another, and showed significant negative paired association with antioxidant enzymes (CAT, POD and SOD), chl and Pn (**Figure 3**). Conversely, the antioxidant enzymes (CAT, POD and SOD), chl and Pn varied proportionally and illustrated paired association in same direction (**Figure 3**). Generally, the correlation analysis has proved strong paired association among all traits, both under control and HA treatments (**Figure 3**). Besides, correlogram further confirm the extent of association of traits significantly changed owe to HA application as compared to zero HA application under salt stress.

**Figure 4:**
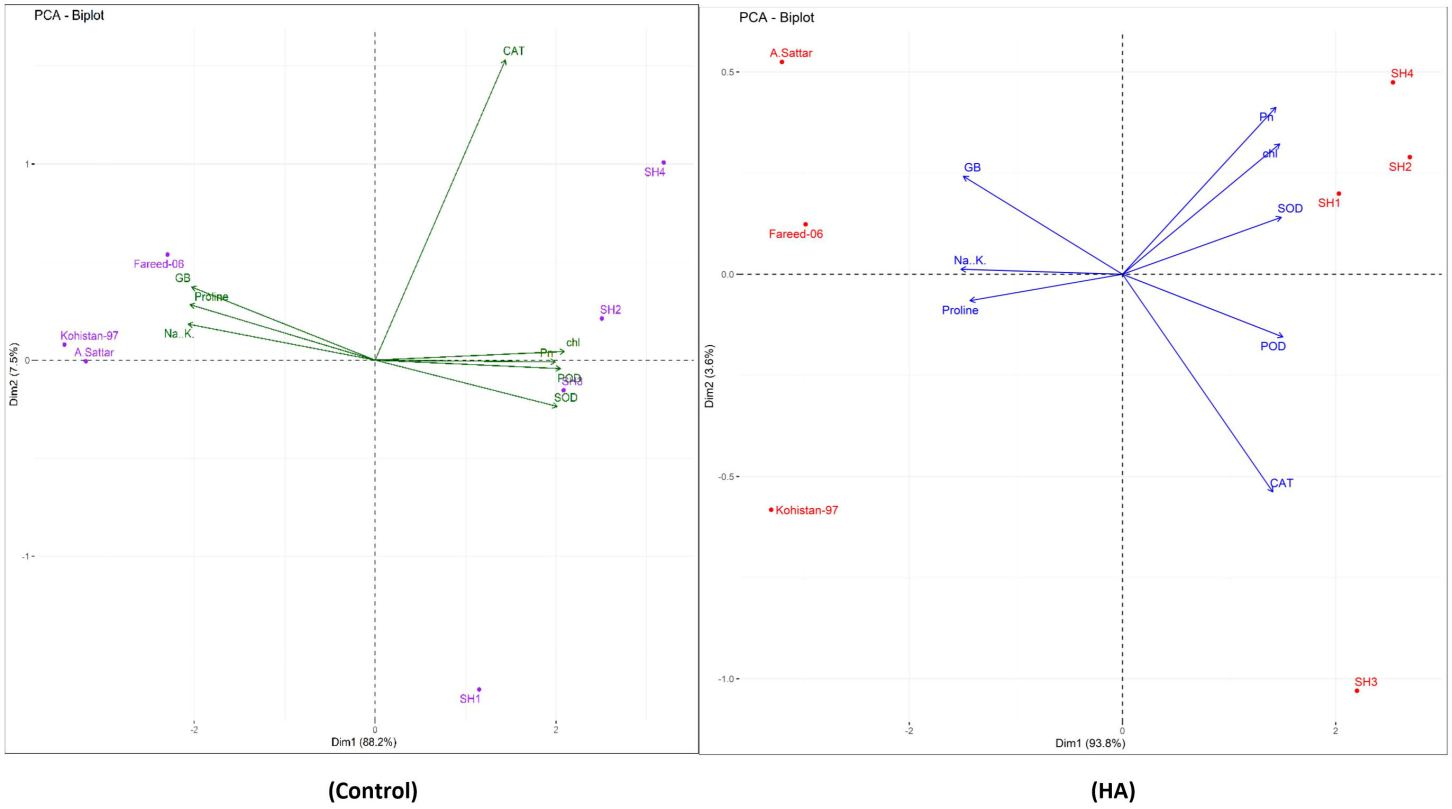
The PCA biplot based upon correlation matrix, indicating the effect of humic HA and control treatment on chlorophyll, photosynthesis, osmolytes (Proline and GB), Na^+^/K^+^ and antioxidant enzymes (SOD, POD and CAT) in salt stressed wheat genotypes. The orientations and closeness of traits vectors differs under HA application as compared to control treatment which confirms the changing association and expression of traits with respect to genotypes categorized into different biplot quadrants. Chl, chlorophyll; Pn, photosynthesis; GB, glycine betaine; SOD, superoxide dismutase; POD, peroxidase; CAT, catalase.

**Figure 5:**
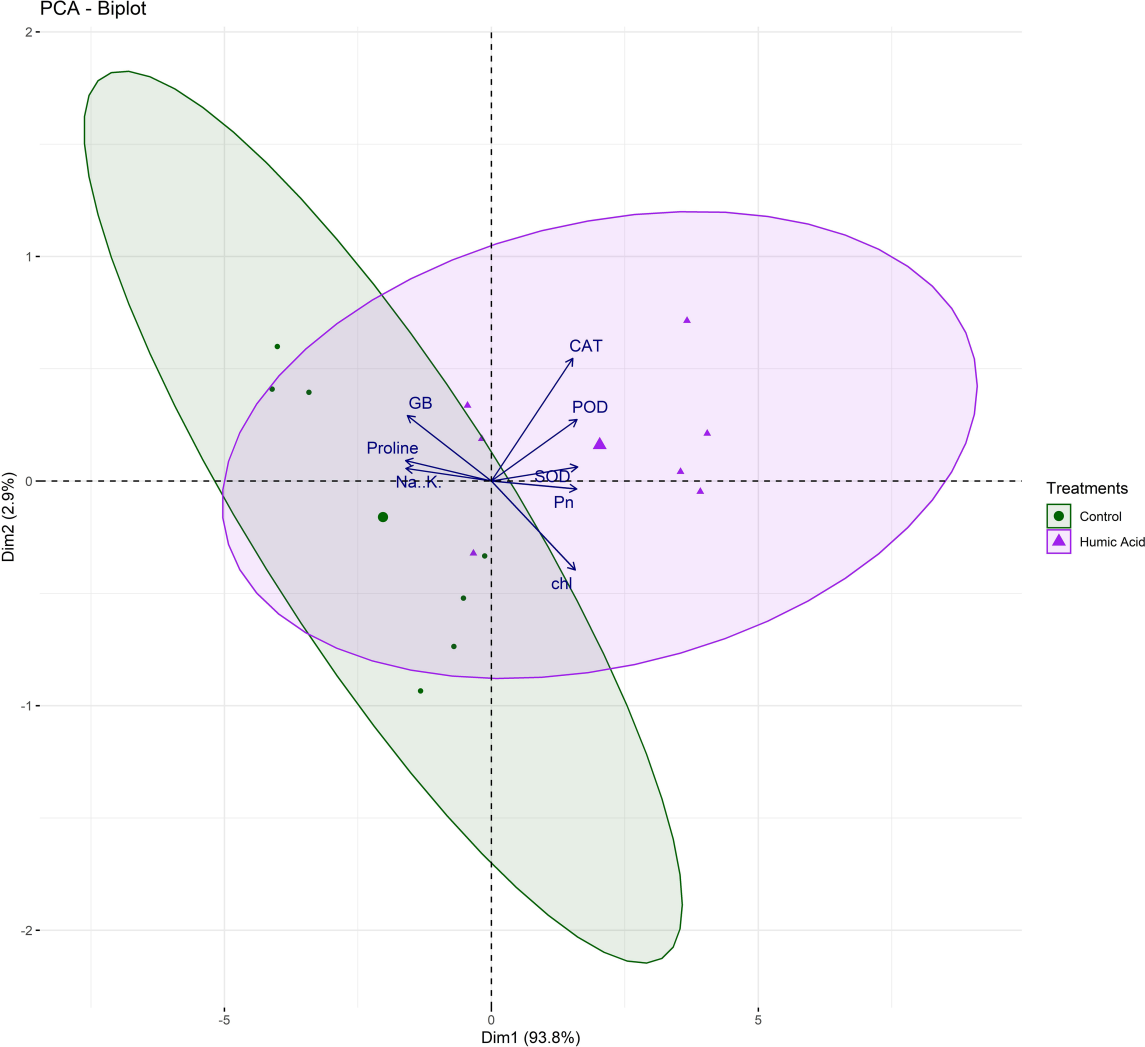
The varying dispersion of ellipses in biplot indicates the strong impact of HA treatment on chlorophyll, photosynthesis and antioxidant enzymes (SOD, POD and CAT), and strong impact of control treatment on osmolytes (Proline, andGB) and Na^+^/K^+^ in salt stressed wheat genotypes. Chl, chlorophyll; Pn, photosynthesis; GB, glycine betaine; SOD, superoxide dismutase; POD, peroxidase; CAT, catalase.

**Figure 6:**
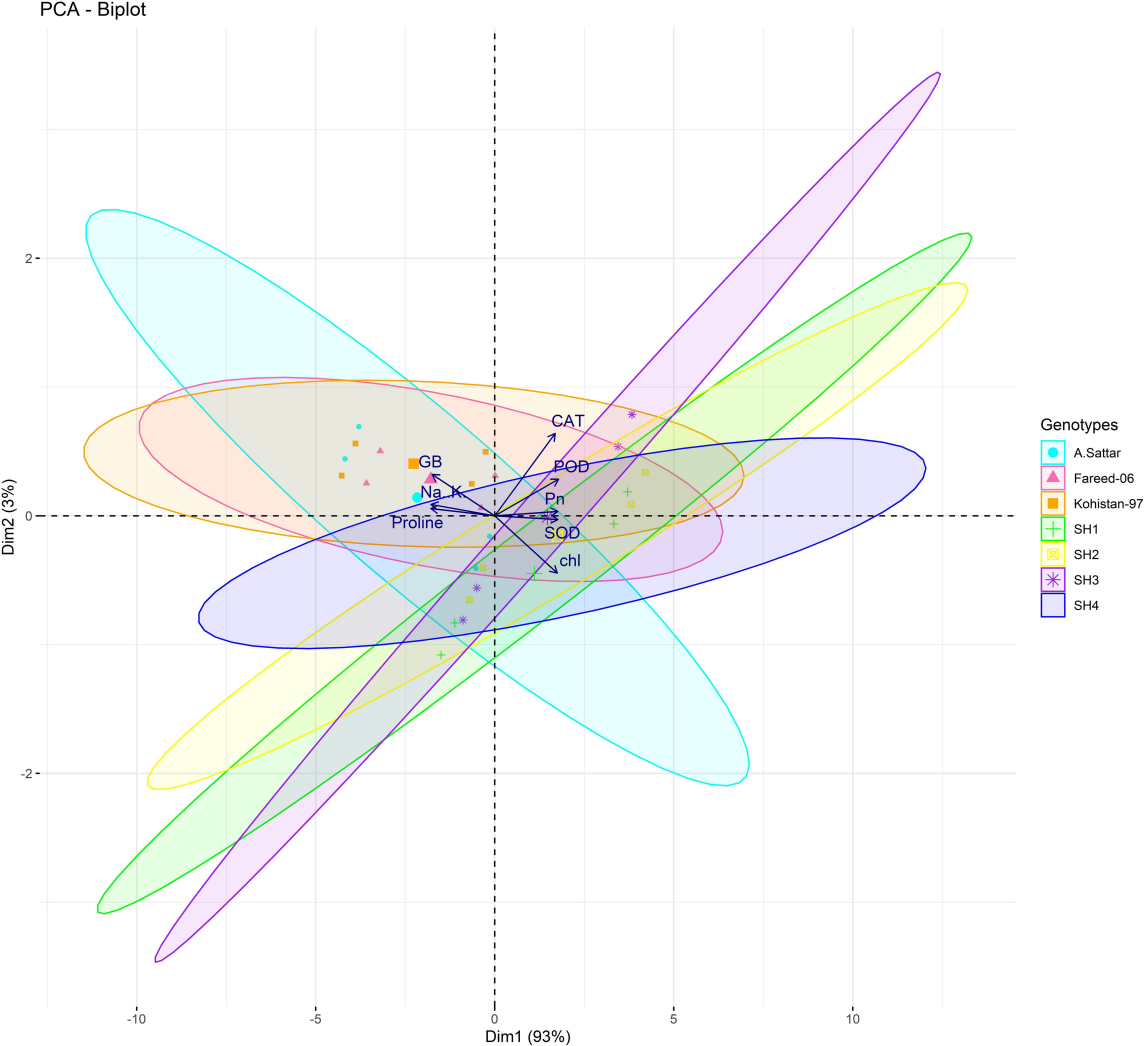
The varying dispersion of ellipses representing salt tolerant SHs (SH1-SH4) and salt susceptible BW in biplot, indicates the high expression of chlorophyll, photosynthesis and antioxidant enzymes (SOD, POD and CAT) in SH, and high expression of osmolytes (Proline, GB) and Na/K in BW genotypes. Chl, chlorophyll; Pn, photosynthesis; GB, glycine betaine; SOD, superoxide dismutase; POD, peroxidase; CAT, catalase.

**Figure 7:**
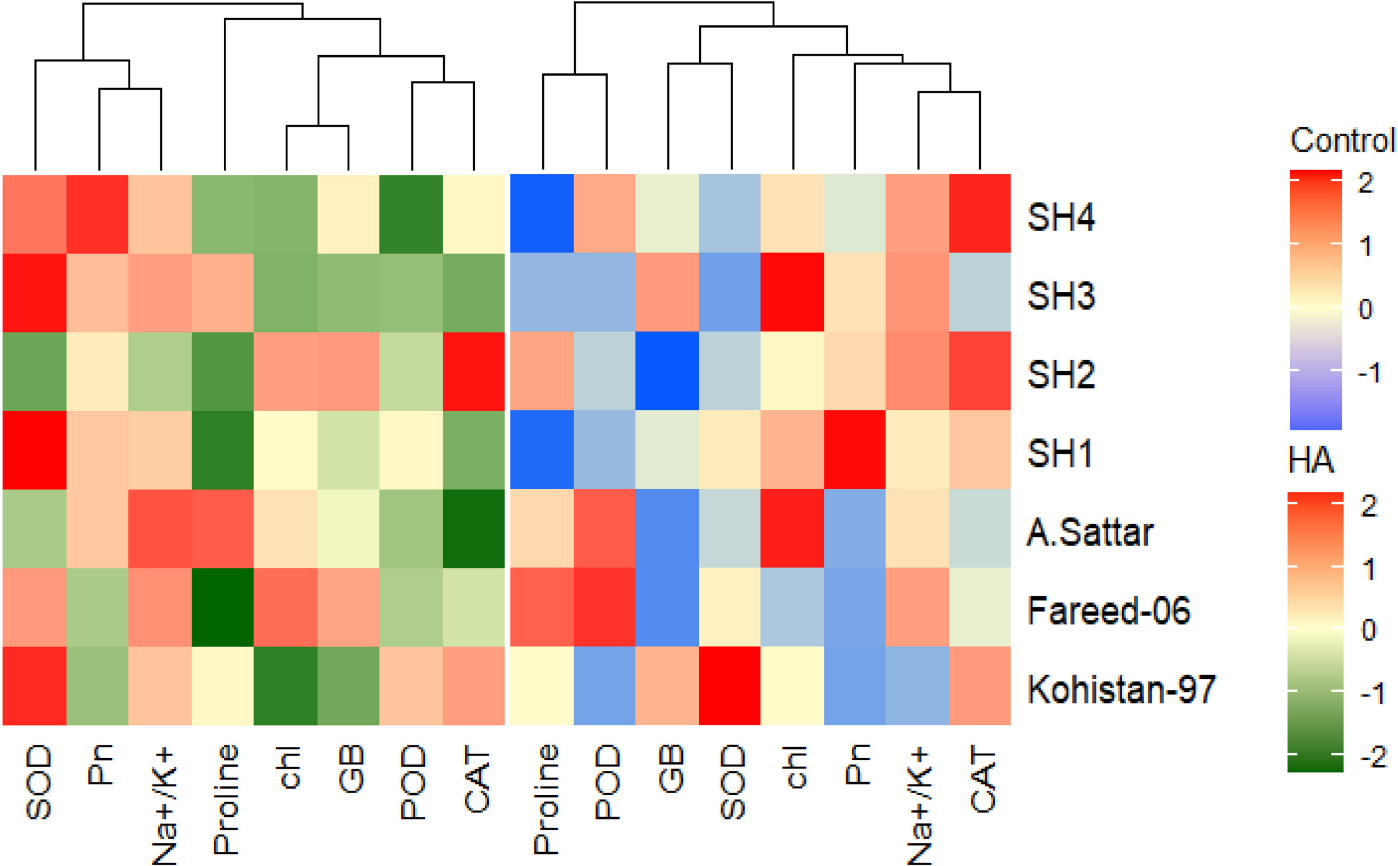
Heatmap categorizing the salt stressed wheat genotypes in terms of the varied trait expression under humic acid and control treatment. The varying colour pattern of bands from light to dark represent the change in the expression of trait from low to high due to the application of HA and control treatment. Chl, chlorophyll; Pn, photosynthesis; GB, glycine betaine; SOD, superoxide dismutase; POD, peroxidase; CAT, catalase.

Besides, principal component analysis (PCA) further endorsed the results from correlation analysis. The biplot of both PCAs under HA and control treatment indicated the different orientation of trait vectors, which confirmed that association and expression of chl, Pn, proline, GB, Na^+^/K^+^ and antioxidant enzymes changed significantly due to HA as compared to control treatment under saline environment (**Figure 4**). Furthermore, PCA confirmed the differential response of each genotype in terms of trait association and expression due to HA application as compared to control as illustrated by their dispersed distribution within PCA biplot (**Figure 4**). In biplot, the SH (SH1 to SH4) genotypes were clustered at one quadrant, while salt susceptible BW genotypes were clustered on another quadrant of biplot. This indicated clear difference in the response of both groups of genotypes to HA under saline environment in terms of trait association (**Figure 4**). Furthermore, the varying impact of HA and control treatment on trait responses were further confirmed by differential positioning of ellipses on PCA biplot (**Figure 5**). The traits including Chl, Pn and antioxidant activity (SOD, POD and CAT) were more strongly expressed under HA treatment, while proline, GB and Na^+^/K^+^ were more strongly expressed under control treatment (**Figure 5**). The PCA biplot indicated that Dim 1 (93.8%) captured most of the variation, isolating the HA and control treatments, while Dim2 (2.9%) contributed slightly, suggesting collectively 96.7% of total variation. Although ellipses showed some overlap, but overall, the treatments showed distinct biochemical responses. Similarly, the different positioning of the ellipses representing the SH (SH1 to SH4) and susceptible BW (Kohistan-97, Fareed-06 and A. Sattar) in PCA biplot has further differentiated the responses of both group of genotypes with respect to the expression of traits (**Figure 6**). The PCA biplot indicated that that Dim1 (93%) captured most of the variation, while Dim2 (3%) added a small part of variation, together comprising the 96% of the total variation. Besides, the merged ellipses of SH group indicated the analogous response of SH (SH1 to SH4) genotypes while the merged ellipses of susceptible BW indicated the analogous response of BW (Kohistan-97, Fareed-06 and A. Sattar) cultivars in terms of trait expression and association. The traits including Chl, Pn and antioxidant activity (SOD, POD andCAT) were more strongly expressed in salt tolerant SH (SH1 to SH4), while proline, GB and Na^+^/K^+^ were more strongly expressed in susceptible BW (Kohistan-97, Fareed-06 and A. Sattar) genotypes (**Figure 6**). The heatmap clustered dendrogram further endorsed the findings from PCA (**Figure 7**). The SH and BW genotypes showed varying trait response under control and HA application as indicated by varying banding pattern. Corresponding to the PCA results the heatmap has divided SH and susceptible BW genotypes into two different groups.

### 3.4 Gene expression analysis

**Figure 8:**
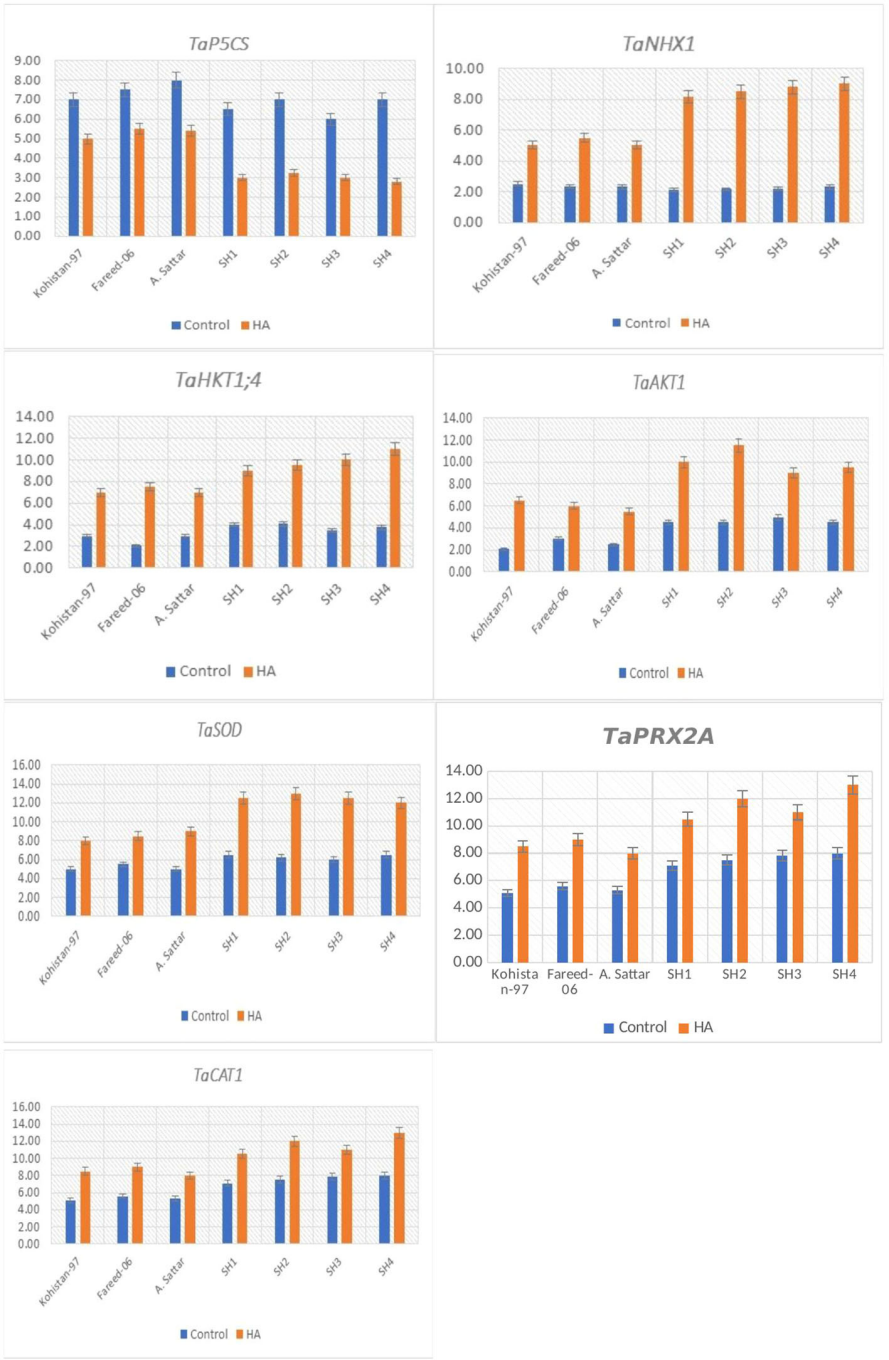
Relative expression analysis of salt stress related genes in susceptible BW and SH wheat genotypes under the application of HA and control treatment.

The gene *TaP5CS* significantly (p ≤ 0.05) downregulated in all salt stressed wheat genotypes due to HA (**Figure 8**). Among all genotypes, the SH (SH1 to SH4) wheat lines showed comparatively low transcript level (1.3 to 2 fold) of TaP5CS as compared to susceptible BW (Kohistan-97, Fareed-06 and A. Sattar) genotypes (**Figure 8**). Besides, the downregulation of *TaP5CS* was in accordance to the declined amount of proline in wheat genotypes grown in saline environment under the application of HA.

The genes *TaNHX1*, *TaHKT1,4* and *TaAKT1* regulating the K^+^-influx and Na^+^- compartmentalization were overexpressed in all salt stressed wheat genotypes from 2 to 4.2 fold, 2.1 to 4 fold, and 1.75 to 3.1 fold respectively, due to the application of HA (**Figure 8**). Overall, the SH (SH1 to SH4) wheat genotypes recorded significantly (p ≤ 0.05) high expression of these genes, that imparted high influx of K^+^, and high efflux of Na^+^, as illustrated by their low Na^+^/K^+^ ratio as compared to susceptible BW (Kohistan-97, Fareed-06 and A. Sattar) genotypes under the application of HA.

The HA significantly (p ≤ 0.05) triggered the expression of *TaSOD* (1.75 to 2.2 fold), *TaCAT1* (1.3 to 2.5 fold) and *TaPRX2A* (1.2 to 1.75 fold) in all salt stressed wheat genotypes (**Figure 8**). Overall, the relative expression of these genes was significantly high in SH (SH1 to SH4) as compared to susceptible BW genotypes. Besides, the increase in transcript level of *TaSOD, TaCAT1* and *TaPRX2A* was in agreement with increasing activity of antioxidant enzymes SOD, CAT and POD.

## 4 Discussion

Salinity is a potential constraint restricting wheat production throughout the world. Salt stress effect plant directly by perturbing physiological and biochemical processes and indirectly by imposing oxidative stress. However, plant biostimulant such as HA induces salt tolerance in plants owe to its potential role in decreasing Na^+^ toxicity, enhancing osmolytic concentrations, lowering Na^+^/K^+^ ratio, and increasing ROS scavenging by speeding up the catalytic activities of antioxidant enzymes. Salt stress significantly decreases the chlorophyll content depending upon the extent of salt tolerance. Various studies have shown that high salt content results in stomatal closure, restricting the CO_2_ fixation, reducing the chlorophyll content which in turn decreases the rate of photosynthesis in plants (El Sabagh et al., 2021; Irshad et al., 2022; Alghabari and Shah, 2021). The humic substances are plant biostimulants and regulate plant physiological processes for sustainable growth particularly under stress (Canellas and Olivares, 2014). Humic acid (HA) protects plant photosynthetic apparatus under various abiotic stresses and improves the chlorophyll and Pn as reported by Shukry et al. (2023). Similarly present study reported an improvement in chlorophyll and Pn in salt stressed wheat genotypes in the presence of HA, however this improvement varied based upon the type of wheat genotype i.e less improvement in susceptible BW, while high improvement in diverse SH (**Figure 1**). Alteration in osmolytic content under salt stress is portrayed as a plant strategical tool to counter the hazards of salt stress (Shah et al., 2017). However, application of HA decreases the concentration of proline, GB and Na^+^ in salt stressed plant based upon their stress tolerance tendency as explicated by Khedr et al. (2022); Shah et al. (2018) and Hamed (2021) respectively. Likewise, Meng et al. (2023) concluded that reduction in proline content of salt stressed plants under HA application confirms the role of HA as a stress reliever, suggesting that plants get rid from osmotic stress, thereby minimizing the need of proline as an osmoprotectant. Likewise, according to Carillo et al. (2008) the HA treatment reduced the GB content salt stressed wheat plants through improving the water status and homeostasis, hence minimizing the need of GB deposition. On the other hand, salt stress induces different types of oxidative stresses within cellular system owe to the production of ROS (Alghabari and Shah, 2021). In this context, HA triggers the plant antioxidant defense system and increases the catalytic activity of antioxidant enzymes to relieve the plant from the damages caused by oxidative stress (Abbas et al., 2022). Correspondingly, in current research the HA reduced the GB and proline content in all salt stressed wheat genotypes. Besides, the SH wheat genotypes illustrated more dramatic reduction in proline and GB as compared to susceptible BW cultivars (**Figure 1**). In the same way, HA enhanced the activity of antioxidant enzymes CAT, POD and SOD in all salt stressed genotypes with high enhancement in SH as compare to BW susceptible genotypes (**Figure 2**).

In present study the HA treatment has reduced the accumulation of osmoprotectants such as proline and GB in salt stressed wheat plants, primarily owe to its role in stress mitigation and physiological stability. Plants usually synthesize these osmolytes under salinity stress in order to alleviate osmotic imbalance and oxidative stress. As HA improves water retention, plant water uptake and soil structure, therefore reducing the need for excessive accumulation of osmolytes (Canellas et al., 2015). Besides, HA triggers the activity of antioxidant enzymes such as SOD, POD and CAT, which significantly reduces the accumulation of ROS, hence declining the stress induced production of GB and proline (Aydin et al., 2012). Additionally, HA promotes ion homeostasis and nutrient uptake, specifically by increasing Na/K selectivity, hence alleviating the ionic toxicity and reducing the necessity for osmoprotectant based stress adaptation (Nardi et al., 2016). Besides, HA treatment also promotes the production of cytokinins, auxins and gibberellins, which alters plant metabolic mode from stress tolerance to growth and development, resulting to a fall is osmolytes deposition (Zhang et al., 2020). Furthermore, HA triggers the chlorophyll and Pn, that further promote plant health and decreasing the plant dependence on stress-induced proline and GB production (Karakurt et al., 2009). As a whole, the HA tendency to counteract osmotic-stress, increase ion-homeostasis, boost antioxidant defense, and induce growth metabolism, explicates the recorded decline in proline and GB concentrations in salt stressed wheat plants.

In past, many studies recorded the potential role of HA in accelerating the vacuolar compartmentalization of Na^+^ and amplifying the influx of K^+^ that reduces the Na^+^/K^+^ and lowered the risk of sodium toxicity under salinity (Mona et al., 2017; Hamed, 2021; Abbas et al., 2022). Complementary, in present study HA lowered the Na^+^/K^+^ ratio in all salt stressed wheat SH and BW genotypes, with maximum reduction in SH as compared to susceptible BW genotypes (**Figure 2**). The physiological and biochemical processes are interlinked and vary in unison when plants are exposed to abiotic stresses (Shah et al., 2017). These processes as a whole determine the plant response to the stress. The plant biostimulants improve the response of plants against stress through strengthening the association of physiological and biochemical indicators of stress as confirmed by the present study (**Figure 3**). Furthermore, Alsamadany (2022) recorded unanimous decline in proline, GB and Na^+^/K^+^ content, while a unanimous rise in the antioxidant activity of enzymes (SOD, POD and CAT), Pn and Chl in salt stressed genotypes due to the application of HA. Correspondingly, current study rectified these findings in salt stressed SH and BW genotypes due to the application of HA (**Figure 4** and **5**). Besides, the salt tolerant SH genotypes had shown the strong association among chl, Pn and the catalytic activity of antioxidant enzymes, while the salt susceptible BW genotype illustrated the strong association among proline, GB and Na^+^/K^+^ratio (**Figure 6**) as reported by Alghabari and Shah (2024). This may be attributed to the role of humic substances in mediating the crosstalk between physiological and biochemical indices of stress tolerance as reviewed by Shah et al. (2024). Moreover, the results from PCA and heatmap further endorsed the potential role of HA in mitigating the effect of salt stress, and high tolerance of SH genotypes to the salt stress based upon the expression of osmolytic, antioxidant and physiological traits (**Figures 4**–**7**). Plants show intraspecific variation in tolerance to abiotic stresses, and response to biostimulants owe their different genetic texture (Shah et al., 2018). In this context, it is important to correlate the physio-chemical mechanisms of stress tolerance to genetic regulators. For instance, the proline producing gene *TaP5CS* overexpress in all salt stressed wheat genotypes to protect the subcellular structure from oxidative stress as reported by Alghabari and Shah (2024). Conversely, Meng et al. (2023) recorded decline in relative expression of *P5CS* gene in salt stressed *Lolium perenne* L due to HA. In parallel, current study reported downregulation of *TaP5CS* along with declined production of proline in all salt stressed wheat genotypes due to supplementation of HA (**Figure 8**). Corresponding to previous results this downregulation was more prominent in SH (SH1 to SH4) genotypes as compared to salt susceptible BW (Kohistan-97, Fareed-06 and A. Sattar) genotypes. Various studies in past rectified the potential role of HA in enhancing the activities of SOD, POD and CAT to reverse the effects of oxidative stress produced by salinity (Abbas et al., 2022; Meng et al., 2023). This is attributed to signaling role of HA that triggers the expression of *TaPOD*, *TaSOD* and *TaCAT* as endorsed by the present study (**Figure 8**). According to Al-Ashkar et al. (2019) less cellular Na^+^/K^+^ and detoxification of ROS are two main elements determining plants tolerance to salt stress. Hence, the antioxidant proteins (SOD, POD, CAT) are essential for ROS scavenging, while the transporter proteins *HKT1* and *NHX1* are mandatory for Na^+^ compartmentalization (El-Hendawy et al., 2017). Besides, Zeeshan et al. (2020) and, Alghabari and Shah (2024) explained the role of overexpressing inward-rectifying K^+^ channel, *TaAKT1*, in speedy influx of K^+^ in salt stressed wheat genotypes to lower the Na^+^/K^+^ ratio. Interestingly, in present study qRT-PCR analysis recorded high relative expression of *TaNHX1* and *TaHKT1,4*, *TaAKT1* along with increased influx of K^+^ in all salt stressed wheat genotypes due to HA, with high expression in SH (SH1 to SH4) as compared to BW (Kohistan-97, Fareed-06 and A. Sattar) susceptible genotypes. In fact, HA enhances the protein abundance of *HKT1, NHX1* and *AKT1* in root stele that triggers the reabsorption of Na^+^ from xylem vessel into neighboring cells, consequently less Na^+^ is translocated to shoot and leaves (Khaleda et al., 2017).

**Figure 9:**
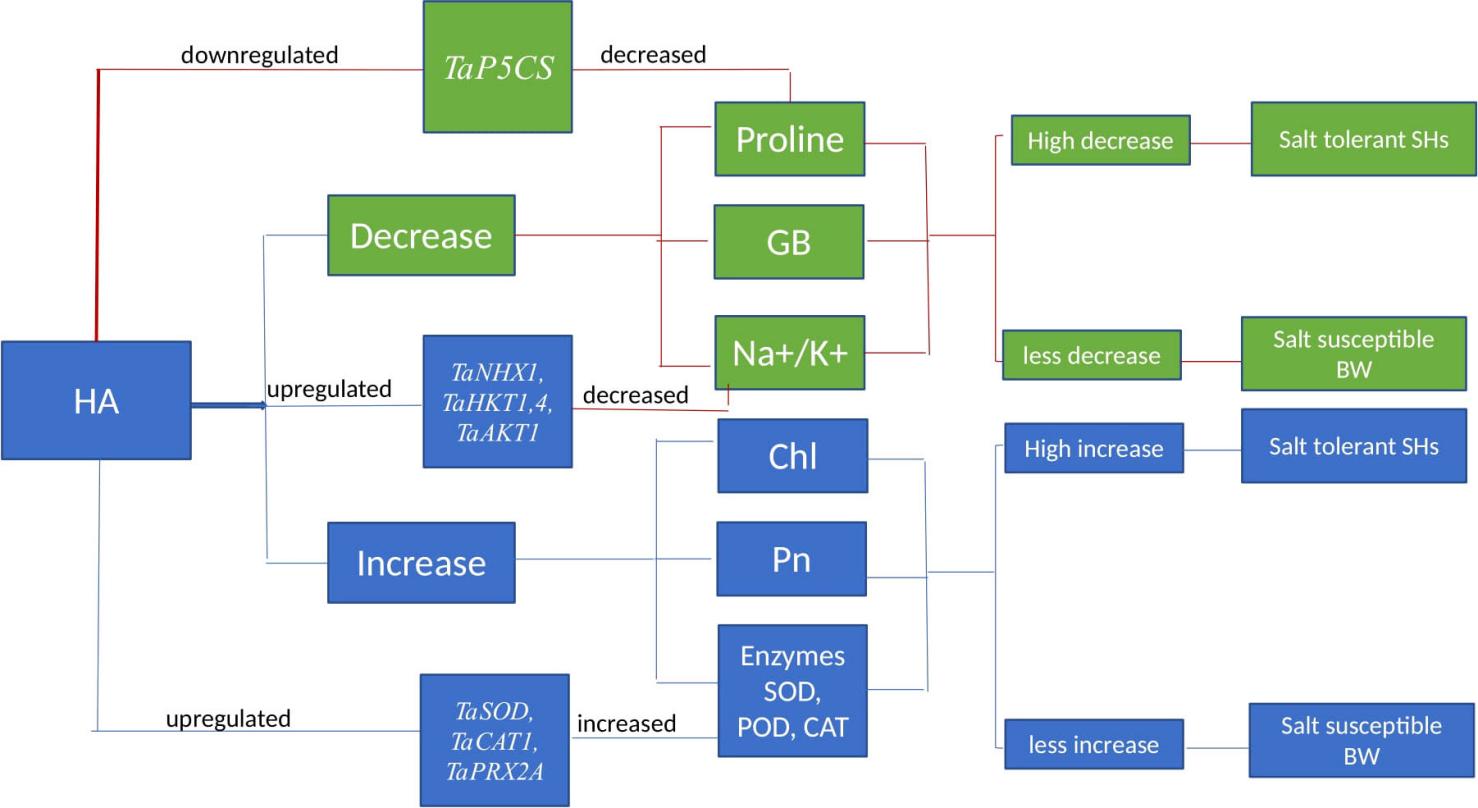
Schematic representation indicating the impact of HA on physiological, biochemical and molecular mechanisms imparting salt stress tolerance. Chl, chlorophyll; Pn, photosynthesis; GB, glycine betaine; SOD, superoxide dismutase; POD, peroxidase; CAT, catalase.

Overall, in present study the HA triggered the physio-chemical and genetic indicators of salinity tolerance in all salt stressed wheat genotypes (**Figure 9**). In addition to role as stress reliever, the scalability and availability of HA for agricultural purposes is significantly important. HA is obtained from naturally occurring humic substances present in lignite, peat, and composted organic matter, making it globally and extensively available (Abbas et al., 2022). However, broad-scale application in progressive and commercial farming requires cost effective production and standardized extraction to ensure agricultural efficacy and sustainability (Shah et al., 2018). HA can significantly increase the soil fertility and reduce the dependency on chemical inputs; however, it cannot completely replace the synthetic fertilizers (Tahir et al., 2022). Although HA helps the plants to develop better tolerance and resilience to environmental stress, but it alone unable to provide sufficient nitrogen, phosphorus and potassium for plant growth. Therefore, the best approach is an integrated nutrient management (INM), mixing HA with organic and synthetic fertilizers, which ensure optimal plant growth and yield. Generally, the SH (SH1 to SH4) genotypes manifested high sensitivity to HA and showed high tolerance to salt stress as compared to susceptible BW (Kohistan-97, Fareed-06 and A. Sattar) genotypes based upon physio-chemical and genetic evaluations. Synthetic hexaploid (SH) wheat has high salt tolerance as compared to domesticated BW owe to wide genetic base inherited from its wild progenitors and less genetic erosion. In fact, the efficient response of SH wheat to salt stress is associated with rich genetic diversity inherited from its least explored D genome as reported by Yang et al. (2014).

## 5 Conclusion

Conclusively, the SH is bridge to transfer alien traits associated with salt tolerance from wild progenitors into elite bread wheat germplasm. The germplasm comparatively evaluated in current study will set a footprint for devising future breeding strategy to impart existing wheat cultivars the tolerance against salt stress.

While present study demonstrated the beneficial effects of HA in mitigating the salt stress under control conditions, there is a dire need for field level validation under varying climatic and soil conditions. The efficiency of HA in the soil of different regions based on soil types, temperature, pH, irrigation practices and organic matter content. Therefore, in future field level trials across different zones are mandatory to test the efficacy of HA in optimizing the wheat production. Furthermore, the large-scale evaluation will provide pragmatic insights into the long-term effects of HA on salt tolerance mechanisms of wheat.

## Data Availability

The original contributions presented in the study are included in the article/supplementary material. Further inquiries can be directed to the corresponding author.
